# Vaccination with EphA2-derived T cell-epitopes promotes immunity against both EphA2-expressing and EphA2-negative tumors

**DOI:** 10.1186/1479-5876-2-40

**Published:** 2004-11-24

**Authors:** Manabu Hatano, Naruo Kuwashima, Tomohide Tatsumi, Jill E Dusak, Fumihiko Nishimura, Karlyne M Reilly, Walter J Storkus, Hideho Okada

**Affiliations:** 1 Department of Neurological Surgery, University of Pittsburgh School of Medicine, 200 Lothrop Street, Pittsburgh, PA, 15213, USA; 2 Department of Surgery, University of Pittsburgh School of Medicine, 200 Lothrop Street, Pittsburgh, PA, 15213, USA; 3 Brain Tumor Program, University of Pittsburgh Cancer Institute, 5117 Centre Avenue, Pittsburgh, PA, 15213, USA; 4 Genetic Modifiers of Tumorigenesis Section, Mouse Cancer Genetics Program, National Cancer Institute at Frederick, West 7th Street at Fort Detrick, PO Box B, Building 560, Room 32-31B, Frederick, MD 21702-1201, USA

**Keywords:** EphA2, dendritic cells, cancer vaccines, melanoma

## Abstract

**Background:**

A novel tyrosine kinase receptor EphA2 is expressed at high levels in advanced and metastatic cancers. We examined whether vaccinations with synthetic mouse EphA2 (mEphA2)-derived peptides that serve as T cell epitopes could induce protective and therapeutic anti-tumor immunity.

**Methods:**

C57BL/6 mice received subcutaneous (s.c.) vaccinations with bone marrow-derived dendritic cells (DCs) pulsed with synthetic peptides recognized by CD8+ (mEphA2_671–679_, mEphA2_682–689_) and CD4+ (mEphA2_30–44_) T cells. Splenocytes (SPCs) were harvested from primed mice to assess the induction of cytotoxic T lymphocyte (CTL) responses against syngeneic glioma, sarcoma and melanoma cell lines. The ability of these vaccines to prevent or treat tumor (s.c. injected MCA205 sarcoma or B16 melanoma; i.v. injected B16-BL6) establishment/progression was then assessed.

**Results:**

Immunization of C57BL/6 mice with mEphA2-derived peptides induced specific CTL responses in SPCs. Vaccination with mEPhA2 peptides, but not control ovalbumin (OVA) peptides, prevented the establishment or prevented the growth of EphA2+ or EphA2-negative syngeneic tumors in both s.c. and lung metastasis models.

**Conclusions:**

These data indicate that mEphA2 can serve as an attractive target against which to direct anti-tumor immunity. The ability of mEphA2 vaccines to impact EphA2-negative tumors such as the B16 melanoma may suggest that such beneficial immunity may be directed against alternative EphA2+ target cells, such as the tumor-associated vascular endothelial cells.

## Background

EphA2 is a member of Eph family of receptor tyrosine kinases comprised of two major classes (EphA and EphB), which are distinguished by their specificities for ligand (ephrin-A and ephrin-B, respectively [1-3]). Recent reports suggest that EphA2 is frequently overexpressed and often functionally dysregulated in advanced cancers, where it contributes to multiple aspects of malignant character. These changes in EphA2 have been observed in a wide array of solid tumors, including melanoma [4,5] and prostate [6], breast [7] and lung [8] carcinomas. Indeed, the highest degree of EphA2 expression among tumors is most commonly observed in metastatic lesions [6,9].

These data suggest that EphA2 may serve as an attractive target for cancer vaccines. In this regard, we have identified five human leukocyte antigen (HLA)-A2 binding and three HLA-DR4-binding peptides derived from EphA2 that are capable of inducing specific, tumor-reactive CD8^+ ^or CD4^+ ^T-cell responses, respectively [10]. A more recent report has identified two additional HLA-A2 restricted T-cell epitopes encoded by EphA2 [11].

These observations and findings support our rationale for near future implementation of EphA2-targeted vaccine clinical trials. For pre-clinical evaluation of EphA2-targeted vaccines, however, there is little information on immune responses against EphA2 in mouse models. Therefore, we hypothesized that identification of mouse T-cell epitopes in mEphA2 would allow us to determine the effect of EphA2-targeted vaccinations in mice bearing tumors. In this study, we examined whether novel T-cell epitope peptides identified in the mEphA2 protein sequence could elicit protective and therapeutic anti-tumor immune responses in murine models. Our results indicate that DC-based vaccines incorporating these peptides elicit effective CTL responses that can inhibit the growth both EphA2+ and EphA2-deficient tumors.

## Materials and methods

### Animals

Female 6–8-week-old C57BL/6 mice were purchased from The Jackson Laboratory (Bar Harbor, ME). Animals were handled under aseptic conditions in microisolator cages within the Central Animal Facility at the University of Pittsburgh per an Institutional Animal Care and Use Committee-approved protocol, and in accordance with recommendations for the proper care and use of laboratory animals.

### Cell Lines and Culture

Glioma cell lines KR129, KR130, KR233 and KR158D were derived from spontaneously arising gliomas in F1 between B6 × CBA (KR129), B6 × SJL (KR130), and C57BL/6 (KR233 and KR158D)-background NPcis mice that express mutations in two tumor-suppressor genes, *Nf1 *and *Trp53 *[12]. B16 melanoma, NPcis-derived glioma cells, GL261 glioma and MCA205 sarcoma (H-2^b^) cell lines were cultured in complete medium (CM) [RPMI 1640 supplemented with 10% heat-inactivated fetal bovine serum, 100 units/ml penicillin, 100 μg/ml streptomycin, and 10 mM L-glutamine (all reagents from Life Technologies, Inc., Grand Island, NY)] in a humidified incubator in 5% CO_2 _at 37°C.

### Generation of DCs in Vitro from Bone Marrow

The procedure used to generate DCs has been previously described [13]. Briefly, C57BL/6 bone marrow cells were cultured in CM supplemented with 1000 units/ml recombinant mouse granulocyte/macrophage colony-stimulating factor and recombinant mouse interleukin-4 (Schering-Plough, Kenilworth, NJ) at 37°C in a humidified, 5% CO_2 _incubator for 7 days. DCs were then isolated at the interface of 14.5% (w/v) metrizamide (Sigma, St. Louis, MO) in CM discontinuous gradients by centrifugation. DCs typically represented >90% of the harvested population of cells based on morphology and expression of the CD11b, CD11c, CD40, CD54, CD80, CD86, and class I and class II MHC antigens as assessed using flow cytometry (data not shown).

### Peptides and immunization

The protein sequences of mEphA2 was obtained from GenBank and analyzed for H-2K^b^-, H-2D^b^-, and I-A^b^-binding binding motifs using BIMAS, and a proteosomal cleavage site prediction system [14], respectively. Peptide sequences that were given high binding scores and predicted proteosomal cleavage sites at the ends of the sequences were chosen [15]. The H-2D^b^-binding mEphA2_671–679 _(FSHHNIIRL), H-2K^b^-binding mEphA2_682–689 _(VVSKYKPM), and I-A^b^-binding mEphA2_30–44 _(LLDFAAMKGELGWLT) epitopes were synthesized using an automated solid-phase peptide synthesizer (Applied Biosystems, Foster City, CA) by the protein synthesis facility at the University of Pittsburgh Cancer Institute, purified (to greater than 95%) by reverse phase HPLC, and characterized for amino acid sequences by mass spectrometry.

Day 7 DCs were pulsed with 10 μM each of the indicated peptides for 4 hours at 37°C, as previously described [13]. Cells were then washed twice with Hank's balanced salt solution (HBSS), with animals receiving injections of the indicated numbers of peptide-pulsed DCs in 0.1 ml HBSS s.c.

### Tumor challenge

In the s.c. model, animals were injected with the indicated numbers of tumor cells in the right flank. Anti-tumor responses were assessed based on comparative longitudinal measurements of tumor area. In the lung metastasis model, animals were injected i.v. with 2 × 10^5 ^B16-BL6 tumor cells on day 0, and they subsequently received s.c. vaccinations of peptide-loaded DCs on days 3, 10 and 17. Animals were sacrificed on day 28 post-tumor injection and analyzed for the assessment of pulmonary metastases by enumerating the number of surface tumor-nodules.

### Western Blotting

Protein lysates isolated from normal mouse brain, spleen, liver, lung, heart, skeletal muscle, and mouse tumor lines were separated by SDS-PAGE, blotted onto nitrocellulose and analyzed for expression of mEphA2 using EphA2 monoclonal antibody (C-20 Ab; Santa Cruz Biotechnology, Inc., Santa Cruz, CA). Blots were imaged on Kodak X-Omat Blue XB-1 film (NEN Life Science Products, Boston, MA) after using horseradish peroxidase (HRP)-conjugated goat anti-rabbit Ig (Biorad, Hercules, CA) and the Western Lighting™ chemiluminescence detection kit (Perkin Elmer, Boston, MA).

### CTL activity assay

Single cell suspensions of SPCs were cultured at 2 × 10^6 ^cells/ml with 2 μg/ml mEphA2_671–679 _or mEphA2_682–689 _in presence of 10 U/ml human IL-2 (Chiron, Emeryville, CA), 50 μM 2-mercaptoethanol (Sigma), and 50 μM N^G ^mono-methyl-L-arginine (Cyclopss, Salt Lake City, UT) in 24 wells plates (Corning, Corning, NY) for 5 days. Specific CTL activity was determined in 4 h ^51^Cr release assays against the indicated target cells, as previously described [16].

### Matrigel plug assay

Eight-week-old mice were injected s.c. twice (days -14 and -7) with DCs loaded with either EphA2-derived peptides (EphA2 _671–679/30–44_) or OVA-derived peptides (OVA _257–264/265–280_). Each animal then received 400 μl of Matrigel (BD Biosciences) supplemented with 400 ng/ml vascular endothelial growth factor (VEGF) in the dorsal area [17]. The animals were sacrificed 10 days later, then the plugs were removed and photographed.

### Statistical analysis

Comparative numbers of lung metastasis, growth of s.c. tumors and T cell responses were compared by Student's *t *test for two samples with unequal variances. Statistical significance was determined at p value <0.05.

## Results

### Expression of mEphA2 in murine tumor cells

**Figure 1:**
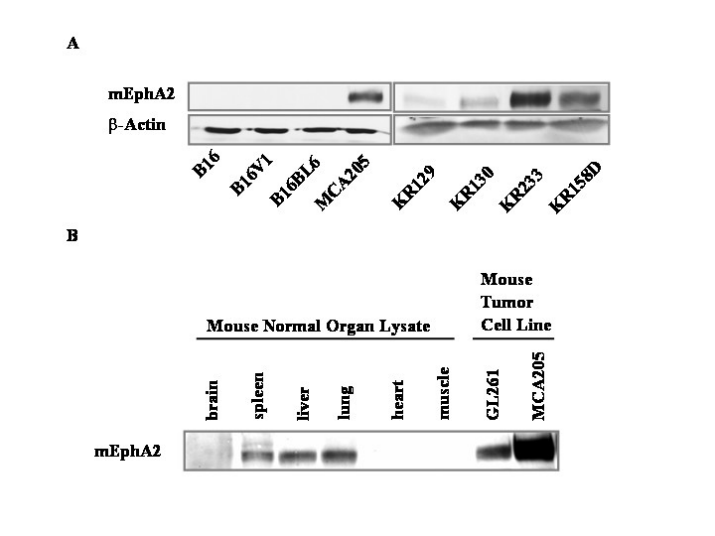
**Expression of EphA2 in murine tumor cell lines and tumor/normal tissues. **(A) Aliquots of protein lysates (10 μg/lane) were analyzed by Western Blotting for expression of mEphA2 protein using a specific monoclonal antibody (C-20 Ab; Santa Cruz Biotechnology, Inc., Santa Cruz, CA). Lysate samples were obtained from mouse tumor lines including the B16, B16V1 and B16BL6 melanomas and the KR129, KR130, KR233 and KR158D glioma cell lines derived from spontaneously developed glioma tissues in NPcis mice. Control β-actin labeling demonstrated equal amount of protein loading in each lane. (B) Protein lysates isolated from normal mouse brain, spleen, liver, lung, heart, skeletal muscle, and the G261 glioma and MCA 205 sarcoma cell lines were separated by SDS-PAGE, blotted onto nitrocellulose and analyzed for expression of mEphA2 using C-20 anti-EphA2 monoclonal antibody.

We evaluated the expression of mEphA2 in H-2^b^-syngeneic murine tumor cell lines and normal organs from C57BL/6 mice by Western Blotting (Figure 1). MCA205 sarcoma, the NPcis mice-derived spontaneously developed glioma cell lines KR129, KR130, KR233 and KR158D (Figure 1A), as well as, the GL261 glioma (Figure 1B) expressed detectable but variable levels of mEphA2 protein. In marked contrast, none of melanoma lines tested, including B16 melanoma and its more metastatic sub-clones B16V1 and B16BL6, expressed detectable levels of mEphA2 (Figure 1A). The control β-actin staining on the same blots demonstrated that equal amount of protein was loaded in each lane. With regard to the normal organs examined, moderate levels of mEphA2 expression were identified in the spleen, liver and lung, whereas no detectable expression was observed in brain, heart and skeletal muscle (Figure 1B).

### Vaccination of mice with synthetic mEphA2 T cell epitopes promotes specific CTL responses

**Figure 2:**
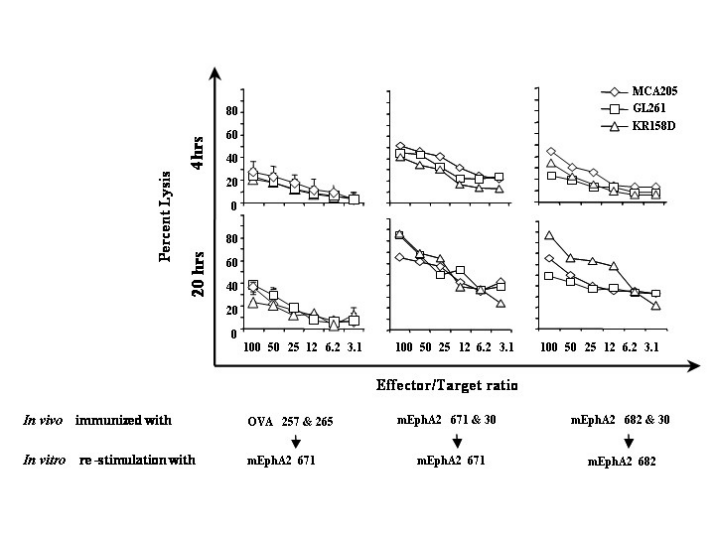
**Immunizations with mEphA2-derived peptides elicit anti-tumor CTLs. **C57BL/6 mice received two cycles of s.c. immunization with 5 × 10^5 ^DCs loaded with mEphA2-derived peptides or control OVA-derived peptides. One week after the second immunization, the animals were sacrificed, and SPCs were re-stimulated *in vitro *with the indicated peptides in the presence of 20 IU/ml hIL-2 for 7 days. Standard 4-hr (upper panel) and 20-hr (lower panel) ^51^Cr-release assays were used to assess cytotoxicity of the responder SPCs against the EphA2+ MCA205, GL261 and KR158D tumor cell lines. Each value represents the average of triplicate determinations for each group. YAC cells were evaluated as non-specific target cells, while EphA2-negative B16 melanoma cells were examined as negative control target cells in all groups, with the lysis of each of these targets constantly less than 10% (data not shown).

We have previously characterized the immunogenicity of the H-2D^b^-binding mEphA2_671–679_, H-2K^b^-binding mEphA2_682–689_, and I-A^b^-binding mEphA2_30–44 _epitopes based on delayed-type hypersensitivity responses in B6 mice (Storkus *et al*., unpublished results). We immunized syngeneic C57BL/6 mice twice s.c. with syngenic DCs loaded with the K^b^-binding and the I-A^b^-binding epitopes, or DCs loaded with the D^b^-binding and the I-A^b^-binding epitopes on a weekly basis. Control animals received DCs loaded with the cancer-irrelevant K^b^-binding OVA_257–264 _and the I-A^b^-binding OVA_265–280 _epitopes [18]. One week after the secondary immunization, the animals were sacrificed, and SPCs were harvested. The SPCs were then stimulated *in vitro *with the MHC class I peptide that was used for *in vivo *immunization, in the presence of 10 U/ml hIL-2, for 7 days. SPCs from control animals immunized with OVA peptides were stimulated *in vitro *with the mEphA2_671–79 _to provide an index for a control, "primary" level of specific CTL induction. CTL activity against mEphA2-expressing, and H-2^b^-syngeneic MCA205 sarcoma, GL261 glioma and KR158D glioma cells were assessed by standard ^51^Cr-release assays (Figure 2). All three mEphA2+ cell lines demonstrated susceptibility to CTLs generated by the immunizations with K^b^- or D^b^-binding mEphA2 derived epitopes. With 20 h – incubation time, the percent lysis by EphA2-induced CTLs increased remarkably in comparison to 4 h-incubation regimen, whereas the lysis by OVA-induced CTLs do not, suggesting that the specific lysis can be more appreciated with the prolonged incubation time (up to 20 h) for the CTLs raised against EphA2-epitopes. B16 melanoma and YAC cells, which were used as negative control target cells, displayed constantly less than 10% of specific lyses in all groups (data not shown). These data support the conclusion that these mEphA2-derived peptides may serve as effective *in vivo *immunogens for anti-tumor T cell activation.

### mEphA2 peptide-pulsed DC-based vaccines effectively protect/treat s.c MCA205 sarcoma and B16 melanoma

**Figure 3:**
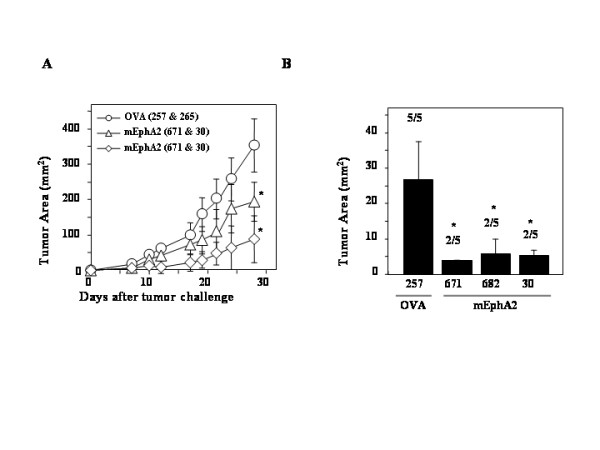
**Immunizations with DCs loaded with Eph-A2 derived peptides inhibit the growth of both EphA2-positive and EphA2-negative tumors. **(A) C57BL/6 mice received 1 × 10^5 ^MCA205 cells s.c. on day 0. These animals were then immunized with 1 × 10^6 ^DCs loaded with the indicated peptides on days 3, 10. Tumor growth was monitored for 28 days following the tumor inoculation (N = 5/group). *P *< 0.05 for both mEphA2_671–679/30–44 _and mEphA2_682–689/30–44 _treatments in comparison to OVA control for the tumor based on a two-tailed Student-t test (*). (B) C57BL/6 mice received s.c. pre-immunizations with 1 × 10^6 ^DCs loaded with either mEphA2_671–679_, mEphA2_682–689_, mEphA2_30–44 _or control OVA_257–264 _on days -14 and -7 (N = 5 /group), followed by s.c. challenge with 5 × 10^4 ^B16 melanoma cells on day 0. On day 14, the size of tumors and the number of animals that had measurable tumors were assessed. P < 0.01 for EphA2_671–679_, EphA2_682–689 _and EphA2_30–44 _treatments in comparison to OVA_257–264 _treated group (*).

We evaluated the efficacy of protective and therapeutic immunizations with mEphA2-derived peptides in s.c. tumor models. These included the mEphA2-positive MCA205 sarcoma and the mEphA2-negative B16 melanoma models. First, animals bearing established s.c. MCA205 tumors received therapeutic vaccinations with mEphA2-derived CTL (H-2K^b ^or H-D^b ^presented) + Th epitope peptides on days 3 and 10 following tumor inoculation (5 animals/group). Control animals received DCs loaded with the OVA-derived CTL + Th epitopes. As displayed in Figure 3A, vaccination with either combination of mEphA2-derived CTL (H-2K^b ^or H-D^b ^presented) + Th epitope peptides significantly inhibited the growth of tumors in comparison to control vaccinations with irrelevant (OVA)-derived T cell epitopes.

We next examined whether vaccination with mEphA2-derived peptides could induce anti-tumor effects against mEphA2-negative tumors, expecting that this would not be the case. Since s.c. B16 melanomas grow aggressively, we employed a pre-immunization model to assess the anti-tumor response. Syngeneic C57BL/6 mice were pre-immunized with either mEphA2_671–679_, mEphA2_682–679_, or mEphA2_30–44 _peptides on days -14 and -7 before s.c. injection with 5 × 10^4 ^B16 cells on day 0 (5 animals/group). The control group received irrelevant OVA_257–264 _instead of mEphA2-derived peptides. Tumor size and the number of animals that had measurable tumors are assessed on day 21 (Figure 3B). All control animals receiving OVA-immunization developed large tumors, whereas, surprisingly, 3 of 5 animals in each of mEphA2-derived peptide-treated groups rejected the tumor growth. Indeed, the average tumor size in each of mEphA2-immunized groups was significantly smaller than the control groups (P < 0.01). As B16 melanoma cells did not express mEphA2 protein *in vitro *(Figure 1A), these data suggest that vaccination with mEphA2-derived peptides may induce *in vivo *CTL activity not only directly against mEphA2 expressed in tumors, but also against mEphA2 expressed by other critical components of tumor-structure, such as tumor vascular endothelial cells. With regard to EphA2 expression *in vivo*, we attempted immunohistochemistry on s.c. B16 tumors to directly validate EphA2 expression. Although deposition of endogenous melanin in B16 tissues posed interference for the substrate-development, we did not observe EphA2-specific staining in s.c. B16 tumor tissues (data not shown).

### EphA2 peptide-pulsed DC-based vaccines protect against/treat B16-BL6 lung metastasis

**Figure 4:**
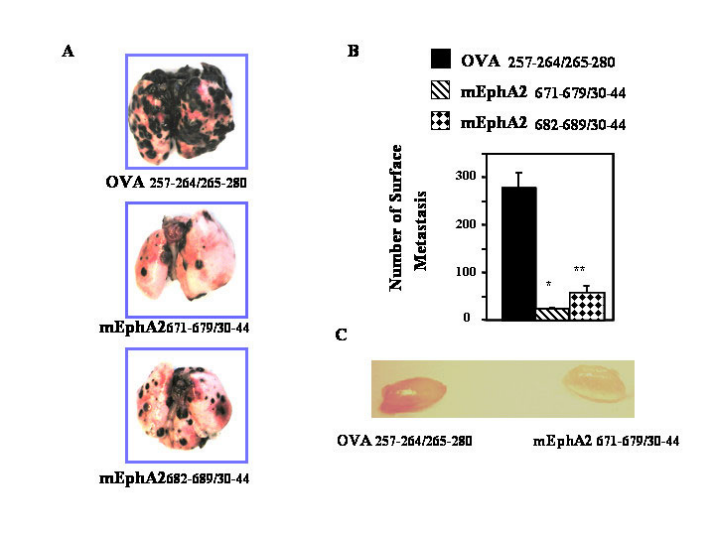
**Inhibition of B16-BL6 lung metastasis and VEGF-induced angiogenesis by s.c. immunizations with DCs loaded with EphA2-derived peptides**. (A and B), C57BL/6 mice received i.v. injections of 2 × 10^5 ^B16-BL6 cells followed by s.c. immunizations with 1 × 10^6 ^DCs loaded with the indicated peptides on days 3, 10 and 17 (N = 4/group). The animals were sacrificed on day 28, with representative macroscopic pictures of the harvested lungs from each group (A) and the numbers of pulmonary surface metastases counted (B) *P *< 0.001 for both mEphA2_671–679/30–44 _(*) and mEphA2_682–689/30–44 _(**) treatments in comparison to OVA control for the number of metastasis based on two-tailed Student-t test. (C), mEphA2-targeted immunizations inhibited the vascular formation in the Matrigel plug assay. Syngeneic mice pre-immunized with control OVA- (left) or EphA2- (right) peptides were injected s.c. with Matrigel containing VEGF. After 10 days, the plugs were removed and photographed. The picture shows representative VEGF-containing plugs excised from total 5 mice/group.

We next evaluated a lung metastasis model using B16-BL6 melanoma cell line. Syngeneic C57BL/6 mice received 2 × 10^5 ^B16-BL6 melanoma cells via tail vein injection. The animals subsequently received s.c. immunizations with 1 × 10^6 ^DCs loaded with mEphA2 CTL (H-2K^b ^or H-D^b ^presented) + Th on days 3, 10 and 17 (5 animals/group). Control animals received DCs loaded with the OVA CTL + Th epitopes. On day 28, the animals were sacrificed, and the lungs harvested from each animal. The number of surface pulmonary metastases and lung weight were measured and compared between the groups. As demonstrated in Figure 4A and 4B, both of mEphA2-immunization regimens resulted in remarkable suppression of B16-BL6 lung metastases in comparison to the OVA-immunized control group in this early-stage treatment model.

VEGF has been implicated to be a major mediator of tumor-angiogenesis [19,20], and EphA2-blockade has been shown to inhibit VEGF-induced angiogenesis [21]. To examine whether immunizations with mEphA2-derived peptides inhibit VEGF-induced angiogenesis, Matrigel plugs containing VEGF were implanted in pre-immunized mice and recovered 10 days later. Macroscopic analysis revealed that the plugs from mice pre-immunized with mEphA2-derived peptides were much paler than those from control mice (Figure 4C). These results suggest that vaccinations with mEphA2-derived T cell epitopes may induce potent anti-tumor responses in mEphA2-deficient tumor models through inhibition of tumor angiogenesis.

## Discussion

In this report, we have demonstrated that mEphA2-derived peptide epitopes can elicit specific CTL responses and anti-tumor response *in vivo*. Of major note, the anti-tumor response was not restricted to mEphA2-postive models, but was also observed in the case of both s.c. and metastatic B16 melanoma models.

While the melanoma cell lines were negative for EphA2 expression *in vitro *as assessed by Western Blotting, it was conceivable that they had become EphA2+ after *in vivo *injection, which could more easily explain the efficacy of the mEphA2 peptide-based vaccinations. However, we were unable to detect mEphA2 expression even in the *in vivo *grown B16 tumors (data not shown), suggesting that the T-cell immunity against mEphA2-derived epitopes suppress the *in vivo *tumor-growth not only through the direct anti-tumor cell mechanisms but also through indirect mechanisms, such as inhibition of tumor-angiogenesis.

The Eph kinases and ephrins play a crucial role in vascular development during embryogenesis and tumor formation [1]. In two independent tumor types, the RIP-Tag transgenic model of angiogenesis-dependent pancreatic islet cell carcinoma and the 4T1 metastatic mammary adenocarcinoma, Ephrin-A1 ligand is expressed in both tumor and endothelial cells, and EphA2 receptor was localized primarily in tumor-associated vascular endothelial cells [3]. Soluble EphA2-Fc or EphA3-Fc receptors inhibit angiogenesis and tumor formation in multiple models [3,22]. Further molecular analyses revealed that the EphA-ephrinA interaction is necessary for VEGF-induced angiogenesis [21,22]. These published observations suggest that immunological targeting of EphA2 may also inhibit tumor growth by suppression of tumor-neoangiogenesis. Immunization against angiogenic-associated antigens selectively expressed on tumor vasculature may provide for a novel strategy to block tumor growth. The feasibility of this approach has been borne out in reports demonstrating that the protein or DNA encoding angiogenic molecules, such as VEGF-R2, can be used as a vaccine to generate effective CTL and antibody responses against tumor vessels, thereby limiting tumor growth and metastasis [19,20]. In this regard, our results with Matrigel assays suggest that immunization of C57BL/6 mice with mEphA2-derived peptides may inhibit VEGF-induced angiogenesis. We are currently confirming these observations with quantitative analyses of vessel formations in the Matrigel plugs.

As expected, based on our Western Blotting, several normal organs examined expressed EphA2 including the spleen, liver and lung. This could provide concern for the potential clinical translation of EphA2-based vaccines, given concerns for autoimmune pathology that might occur in these anatomic sites. However, despite the effective induction of antigen-specific CTLs that are capable of mediating DTH-type reactivity (unpublished data) and anti-tumor responses *in situ*, our careful observation of EphA2-vaccinated animals at autopsy did not reveal any evidence for tissue infiltration by inflammatory leukocytes or the destruction of EphA2+ tissues.

In our assessment of CTL and anti-tumor response, in most cases, we employed both MHC class I and II associated peptides in our treatment regimens based on our previous study demonstrating that the combination of MHC class I and II-restricted OVA-immuno-epitopes induced higher levels of anti-tumor efficacy than MHC class I-restricted OVA-epitopes solely [18]. With regard to the immunogenicity of each mEphA2-derived peptide, our data with preimmunizations against s.c. B16 challenge indicated that each of mEphA2 _671–679_, mEphA2 _682–689 _and mEphA2 _30–44 _could induce anti-tumor response. It was noteworthy that mEphA2 _30–44_, which was found to bind I-A^b ^and induce DTH and specific CD4+ T cell responses, also suppressed the growth of s.c. (MHC class II-deficient) B16 melanomas as a single agent. This may suggest that neovessels in the B16 microenvironment are MHC class II+ either constitutively or under inflammatory conditions under which IFN-γ is elaborated, that B16 melanomas become class II+ after *in vivo *transfer or upon provision of IFN-γ *in situ*, or that the mEphA2 _30–44 _Th peptide also contains an embedded CTL epitope. We are currently assessing each of these possibilities.

It is also intriguing to determine whether there is a correlation between expression levels of mEphA2 in the target cells and their susceptibility to CTLs raised against EphA2-derived peptides. Our preliminary data indicated EphA2 expression levels did not simply correlate with the CTL susceptibility, suggesting other factors, such as MHC class I expression and intracellular anti-apoptotic proteins, may contribute in the CTL-mediated cytotoxicity (data not shown). To address this issue, our ongoing studies with human glioma cells employ small inhibitory RNA for EphA2 to determine whether specific inhibition of EphA2-expression affects the CTL response.

## Conclusions

Although the precise mechanisms that underlie the anti-tumor efficacy of EphA2-based vaccines remain to be elucidated, our results demonstrate that the mEphA2-derived epitopes may represent important vaccine candidates for the development of clinical trials for the treatment of (both EphA2+ and EphA2-negative) cancers.

## List of abbreviations

s.c., subcutaneous; i.v., intravenous; CTL, cytotoxic T lymphocyte; Th, T helper; DCs, dendritic cells; APCs, antigen-presenting cells; OVA, ovalbumin; CM, complete medium; HBSS, Hank's balanced salt solution; PBS, phosphate buffered saline; SPCs, splenocytes; HLA, human leukocyte antigen; ELISPOT, enzyme-linked immuno-spot; DTH, delayed-type hypersensitivity; VEGF, vascular endothelial cell growth factor

## Disclosure of competing interests

The author(s) declare that they have no competing interests.

## Authors' contributions

NK carried out the Western Blotting and immuno-assays. NK, JED, TT, MH and FH carried out assessment of in vivo anti-tumor response and preparation of peptide-pulsed DCs. WJS participated in peptide identification, the design of the study and critical review of data and the manuscript. HO conceived of the study, and participated in its design and coordination. All authors have read and approved the final manuscript.
